# Safety and efficacy of non–vitamin K antagonist oral anticoagulants compared with well-controlled warfarin in Thai patients with atrial fibrillation

**DOI:** 10.2478/abm-2022-0016

**Published:** 2022-06-30

**Authors:** Janekij Yamkasikorn, Komsing Methavigul

**Affiliations:** Department of Cardiology, Central Chest Institute of Thailand, Nonthaburi 11000, Thailand

**Keywords:** atrial fibrillation, factor Xa inhibitors, hemorrhage, non–vitamin K antagonist oral anticoagulants, warfarin

## Abstract

**Background:**

In trials of patients with atrial fibrillation (AF), non–vitamin K antagonist oral anticoagulants (NOACs) were not inferior to warfarin for thromboembolic and bleeding events. However, there are scant data comparing the efficacy and safety of NOACs in patients with AF with that of well-controlled warfarin treatment in such patients.

**Objectives:**

To compare total bleeding and thromboembolic events in patients with AF who received NOACs, with the same events in those who received well-controlled warfarin treatment.

**Methods:**

We used retrospective data from patients with AF who received NOACs or well-controlled warfarin at the Central Chest Institute of Thailand from January 2017 to December 2019. The primary outcome was total bleeding or thromboembolic events or both. The secondary outcome was all-cause mortality, total bleeding events including major or minor bleeding, and thromboembolic events including ischemic stroke or systemic embolization.

**Results:**

We included data from 180 patients with AF, 90 who received NOACs and 90 who received well-controlled warfarin. The average time in the therapeutic range for those who received warfarin was 84.9% ± 9.8%. The patients who received well-controlled warfarin had more frequent thromboembolic or total bleeding events or both than those who received NOACs (odds ratio [OR] 3.17; 95% confidence interval [CI] 2.27–4.07; *P* = 0.01). There were more minor bleeding events in those who received well-controlled warfarin (OR 3.75; 95% CI 2.79–4.71; *P* = 0.01). However, there was no significant difference in thromboembolic events, major bleeding, or all-cause mortality between the 2 groups.

**Conclusions:**

Thai patients with AF who received NOACs had less thromboembolic or total bleeding events than those who received well-controlled warfarin treatment.

Atrial fibrillation (AF) is an arrhythmic disorder commonly observed in the elderly [1]. Many patients with AF have multiple comorbidities, such as hypertension, diabetes mellitus, coronary artery disease, congestive heart failure, chronic kidney disease, and valvular heart disease. Acute ischemic stroke is catastrophic sequelae in patients with AF. AF is associated with a 5-fold increased risk for ischemic stroke and a 2-fold increased risk for mortality [2].

Oral anticoagulants (OAC) are recommended to prevent thromboembolic events in patients with AF and high thromboembolic risk (CHA_2_DS_2_-VASc score of ≥1 in men and ≥2 in women) according to international standard clinical practice guidelines. OAC can reduce the incidence of ischemic stroke and overall mortality. Previous studies have shown that the incidence of ischemic stroke and mortality in patients with AF receiving OAC accounted for approximately 1.5% per year and 3% per year, respectively [3].

Several studies have demonstrated that non–vitamin K antagonist oral anticoagulants (NOACs) are not inferior to warfarin regarding thromboembolic and bleeding events. Moreover, NOACs are convenient to use and have evidence for less frequent intracranial hemorrhage than warfarin. However, the incidence of gastrointestinal bleeding was not lower than that in the warfarin group [4, 5, 6, 7]. A previous NOAC trial has shown that the use of NOACs was associated with lower thromboembolism, major bleeding, and all-cause death than warfarin in octogenarian Asian patients with AF. However, patients taking warfarin had a mean time in the therapeutic range (TTR) of just 49% [8]. Moreover, efficacy and safety of NOACs was studied in patients with AF and hypertrophic cardiomyopathy, but the investigators did not report the TTR in the warfarin group [9]. In any case, there are scant data comparing the efficacy and safety of NOACs in patients with AF with that of well-controlled warfarin treatment (TTR >65%).

We conducted a retrospective observational study to compare the total bleeding or thromboembolic events or both in patients with AF who received NOACs and the same events in those who received well-controlled warfarin treatment.

## Methods

We used retrospective data from Thai patients with AF aged ≥18 years who received OACs at the Central Chest Institute of Thailand from January 2017 to December 2019. We excluded patients with severe chronic kidney disease (creatinine clearance <30 mL/min/1.73 m^2^), rheumatic mitral stenosis, mechanical prosthetic heart valve, thrombocytopenia (platelet counts <100,000/mm^3^), heparin-induced thrombocytopenia, myeloproliferative disorders (essential thrombocythemia, chronic myeloid leukemia, polycythemia vera, agnogenic myeloid metaplasia), hyperviscosity syndrome, discontinuation of OAC due to invasive procedures or surgery more than 3 days, and taking warfarin with TTR <65%. The patients included where chosen using convenience sampling following the hospital number of each patient, which was found from a diagnostic code search and were divided into 2 groups: a group receiving NOACs and a group receiving well-controlled warfarin treatment. Demographic data including sex, age, medical history, CHA_2_DS_2_-VASc score, HAS-BLED score, SAMe-TT_2_R_2_ score, previous treatment and medications, thromboembolic events, bleeding events, and all-cause mortality were collected to cover a period of 12 months. TTR was calculated using the method described by Rosendaal et al. [10]. The primary outcome was total bleeding or thromboembolic events or both. Total bleeding events included major or minor bleeding. Major bleeding events were defined following the International Society on Thrombosis and Haemostasis (ISTH) definition for major bleeding [11]. Minor bleeding events were defined as those involving bleeding other than ISTH-defined major bleeding. Thromboembolic events included ischemic stroke or systemic embolization. The secondary outcome was all-cause mortality, total bleeding events including major or minor bleeding, and thromboembolic events including ischemic stroke or systemic embolization. This study was conducted in compliance with the Declaration of Helsinki and its contemporary amendments (2013) and reported following the recommendations of the RECORD and STROBE statements [12, 13]. The Human Research Ethics Committee of the Central Chest Institute of Thailand approved the study protocol (approval No. 018/2563).

### Statistical analysis

We specified 0.05 for type I error and 0.20 for type II error with 80% power. The estimated bleeding or thromboembolic events in the well-controlled warfarin and NOACs groups were 0.29 and 0.10, respectively [14, 15]. We compared 2 independent proportions using a chi-square test and estimated a required sample size of 180 patients, including an allowance for missing data [16, 17, 18].

We used descriptive statistics for analysis of demographic and clinical data. The categorical data are presented as numbers and percentage. The continuous data are presented as mean and standard deviation (SD) and were analyzed using an independent *t* test if they were normally distributed. The continuous data are presented as median and interquartile range (IQR) and were analyzed using a Mann–Whitney *U* test if they were skewed. We compared primary and secondary outcomes between the NOAC- and well-controlled warfarin-treated groups using a chi-square or Fisher exact test. *P* < 0.05 was considered significant.

## Results

We chose equal-sized groups of patients with AF who had received NOACs or well-controlled warfarin treatment and who were eligible for our study by convenience sampling following a sample size calculation. Of the 180 AF patients receiving OAC, 90 received NOACs and 90 received well-controlled warfarin treatment. Nearly 60% of all patients were male. The average age of all patients was 72.8 ± 10.1 years. The average CHA_2_DS_2_-VASc and HAS-BLED scores were 4 and 2, respectively. The average TTR in the group receiving well-controlled warfarin was 84.9% ± 9.8%. Most patients with AF receiving NOACs were prescribed rivaroxaban and apixaban (62.2%). More patients were taking nonsteroidal anti-inflammatory drugs (NSAIDs) in the NOACs group than in the well-controlled warfarin group. Baseline characteristics are shown in **Table 1**.

**Table 1:** Baseline characteristics of Thai patients with AF

**Demographic data**	**Total (n = 180) n (%)**	**Warfarin (n = 90) n (%)**	**NOACs (n = 90) n (%)**	***P***
Age (years), mean ± SD	72.8 ± 10.1	71.3 ± 10.2	74.3 ± 9.8	0.04^*^
Male sex	107 (59)	48 (53)	59 (66)	0.10
Medical welfare				
Affiliation reimbursement	122 (68)	40 (44)	82 (91)	<0.01^*^
Universal healthcare coverage	43 (24)	40 (44)	3 (3)	
Social security scheme	3 (2)	3 (3)	0 (0)	
Self-payment	12 (7)	7 (8)	5 (6)	
Medical history				
Diabetes	50 (28)	23 (26)	27 (30)	0.51
Hypertension	162 (90)	79 (88)	83 (92)	0.32
Dyslipidemia	139 (77)	69 (77)	70 (78)	0.86
Coronary artery disease	36 (20)	14 (16)	22 (24)	0.14
Peripheral artery disease	0 (0)	0 (0)	0 (0)	–
Previous myocardial infarction	24 (13)	13 (14)	11 (12)	0.66
Previous ischemic stroke or TIA	34 (19)	14 (16)	20 (22)	0.25
History of intracranial bleeding	2 (1)	0 (0)	2 (2)	0.50
History of heart failure	74 (41)	43 (48)	31 (34)	0.07
Cirrhosis	1 (1)	0 (0)	1 (1)	–
Chronic kidney disease	54 (30)	28 (31)	26 (29)	0.78
Smoking, n (%)	48 (28)	23 (26)	25 (29)	0.66
eGFR (mL/min/1.73 m^2^), median (IQR)	70.75 (58.0, 85.8)	70.72 (57.3, 85.1)	70.75 (58.2, 87.0)	0.87
CHA_2_ DS_2_-VASc score, median (IQR)	4 (3, 5)	4 (3, 5)	4 (3, 5.3)	0.30
HAS-BLED score, median (IQR)	2 (1, 2)	1 (1, 2)	2 (1, 2)	<0.01^*^
SAMeTT_2_ R_2_ score, median (IQR)	4 (3, 4)	4 (3, 4)	4 (3, 4)	0.70
0–2	15 (9)	7 (8)	8 (9)	0.75
≥3	157 (91)	80 (92)	77 (91)	
Time in the therapeutic range (%), mean ± SD		84.9 ± 9.8	–	–
Antiplatelets	10 (6)	7 (8)	3 (3)	
Aspirin	3 (2)	2 (2)	1 (1)	–
P2Y_12_ inhibitors	7 (4)	5 (6)	2 (2)	0.44
OAC				
Warfarin		90 (100)	–	–
Rivaroxaban		–	30 (33)	–
Apixaban		–	26 (29)	–
Dabigatran		–	17 (19)	–
Edoxaban		–	17 (19)	–
NSAIDs	6 (3)	0 (0)	6 (7)	0.03^*^
Proton pump inhibitors	51 (28)	23 (26)	28 (31)	0.41

* *P* < 0.05 indicates statistical significance.

AF, atrial fibrillation; SD, standard deviation; NOACs, non–vitamin K antagonist oral anticoagulants; TIA, transient ischemic attack; eGFR, estimated glomerular filtration rate; IQR, interquartile range, NSAIDs, nonsteroidal anti-inflammatory drugs; OAC, oral anticoagulants.

**Figure 1:**
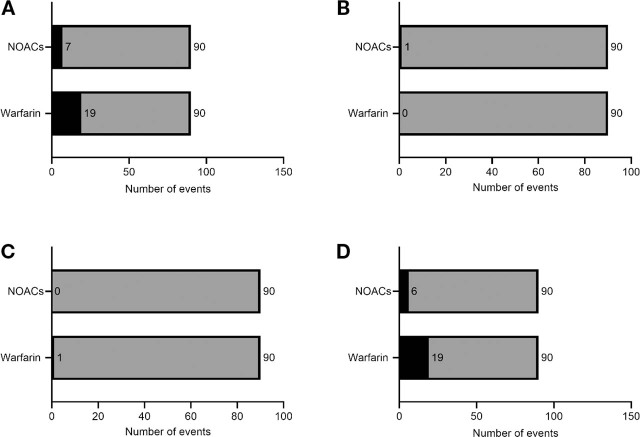
Comparison of total bleeding or thromboembolic events or both (A), thromboembolic events (B), major bleeding (C), and minor bleeding (D) between 90 patients with AF who received NOACs and 90 patients with AF who received well-controlled warfarin (TTR > 65%) treatment. Black bars indicate the presence of events, and gray bars indicate the absence of events. AF, atrial fibrillation; TTR, time in the therapeutic range; NOACs; non–vitamin K antagonist oral anticoagulants.

The group that received well-controlled warfarin treatment had more thromboembolic or total bleeding events or both than the group that received NOACs (odds ratio [OR] 3.17; 95% confidence interval [CI] 2.27–4.07; *P* = 0.01). There were more minor bleeding events in patients who received well-controlled warfarin (OR 3.75; 95% CI 2.79–4.71; *P* = 0.01). However, there was no difference in thromboembolic events, major bleeding, or all-cause mortality between the 2 groups (**Table 2** and **Figure 1**).

**Table 2:** Primary and secondary outcomes of NOAC and well-controlled warfarin treatment in patients with AF

**Outcomes**	**Warfarin (n = 90) n (%)**	**NOACs (n = 90) n (%)**	**OR (95% CI)**	***P***
Primary outcome: total bleeding or thromboembolic events or both	19 (21)	7 (8)	3.17 (2.27–4.07)	0.01^*^
Secondary outcome				
Thromboembolic events or major bleeding or both	1 (1)	1 (1)	1.00 (−1.78 to 3.78)	>0.99
Thromboembolic events	0 (0)	1 (1)	–	–
Major bleeding	1 (1)	0 (0)	–	–
Minor bleeding	19 (21)	6 (7)	3.75 (2.79–4.71)	0.01^*^
Bleeding per gum	2 (2)	1 (1)	2.02 (−0.40 to 4.44)	>0.99
Bruising	12 (13)	5 (6)	2.61 (1.53–3.69)	0.047
Hematuria	3 (3)	2 (2)	1.51 (−0.29 to 3.31)	>0.99
Epistaxis	1 (1)	0 (0)	–	–
Subconjunctival hemorrhage	2 (2)	0 (0)	–	–
All-cause mortality	0 (0)	0 (0)	–	–

* *P* < 0.05 indicates statistical significance.

NOACs, non–vitamin K antagonist oral anticoagulants; OR, odds ratio; CI, confidence interval.

## Discussion

Previous randomized controlled trials have demonstrated that in patients with AF, NOACs were not inferior to treatment with warfarin when considering stroke or systemic embolization or both and major bleeding events [4, 5, 6, 7]. However, a limitation of previous NOAC trials was the poor quality of anticoagulation control in the warfarin arm; a mean TTR <65% was observed in these trials [4, 5, 6, 7]. Moreover, the GARFIELD-AF registry has shown that patients receiving a vitamin K antagonist (66.7% of patients took warfarin) having a TTR <65% had more stroke or systemic embolism, major bleeding, and all-cause mortality than those with a TTR ≥65% [19].

To our knowledge, this is the first study to find that Thai patients with AF who received NOACs had significantly less frequent thromboembolic or total bleeding events or both than those who received well-controlled warfarin treatment, which is consistent with previous reports [14, 15]. In patients with NOACs, the decreased primary outcome was driven by a lower minor bleeding event rate than in patients with well-controlled warfarin, while the frequency of thromboembolic and major bleeding events was comparable between the groups.

The patients with AF under well-controlled warfarin treatment had more antiplatelets than those treated with NOACs. This might lead to increased minor bleeding in those with well-controlled warfarin treatment. However, the patients receiving NOACs also had a history of NSAIDs use, while those under well-controlled warfarin treatment had no history of receiving NSAIDs. This implies that NOACs may have a greater effect in lowering bleeding risk in patients who use NSAIDs than in those who receive warfarin. To date, COX-2 inhibitor use is widespread in many countries and may be used in patients who receive NOACs because of physicians' concerns about gastrointestinal bleeding from conventional NSAIDs, which may manifest as occasional instances of minor bleeding. The possibility of other effects should be clarified.

There are several limitations to the present study. First, we used a relatively small sample size, resulting in a low frequency of thromboembolic and major bleeding events between the NOACs and well-controlled warfarin groups. Second, this study was retrospective, and there may be discrepancies and incompleteness of the information provided, and causality cannot be demonstrated. Third, the patients' baseline characteristics between the 2 groups were different, leading to confounding factors such as age, HAS-BLED score, and use of NSAIDs. A propensity score matching analysis should be conducted to reduce selection bias, and outcomes should be analyzed for competing risk. However, because of the limited sample size, the baseline characteristics of these study patients cannot be reanalyzed. Moreover, simple random sampling could be potentially vulnerable to sampling error because it was not stratified following confounding factors. The patients in this trial were of Thai ancestry, limiting generalizability. Nevertheless, this study was the first, to our knowledge, to show the lower frequency of thromboembolic or total bleeding or both in patients with AF who received NOACs than in those under well-controlled warfarin treatment. Moreover, most patients with AF in the present study had a SAMe-TT_2_R_2_ score of ≥3, implying that NOACs resulted in fewer thromboembolic or total bleeding events or both than well-controlled warfarin treatment, despite a high SAMe-TT_2_R_2_ score.

## Conclusion

Thai patients with AF who received NOACs had fewer thromboembolic or total bleeding events or both than those who received well-controlled warfarin treatment. However, there was no difference in the frequency of thromboembolic events and major bleeding between the 2 groups.
